# Change in Nutrition and Lifestyle in the Eastern Mediterranean Region: Health Impact

**DOI:** 10.1155/2012/436762

**Published:** 2012-06-06

**Authors:** Abdulrahman O. Musaiger, Hazzaa M. Al-Hazzaa, Hamed R. Takruri, Najat Mokhatar

**Affiliations:** ^1^Nutrition and Health Studies Unit, Deanship of Scientific Research, University of Bahrain, Sukhair, Bahrain; ^2^Arab Center for Nutrition, P.O. Box 26923, Manama, Bahrain; ^3^Exercise Physiology Laboratory, King Saud University, P.O. Box 2458, Riyadh 11451, Saudi Arabia; ^4^Department of Nutrition and Food Technology, Faculty of Agriculture, University of Jordan, Amman 11942, Jordan; ^5^Unite Mixte de Researche en Nutritiont Alimentation, URAC 39, Center National L'Energie, des Sciences et Techniques Nucléaires, Universite Ibn Tofail, Kenitra, Morocco

According to World Health Organization, the Eastern Mediterranean Region (EMR) refers to all Arab countries, excluding Algeria, in addition to Iran, Pakistan, and Afghanistan. These countries have faced marked changes in their demographic, socioeconomic, and health status during the last thirty years. These changes have been reflected in changes in the lifestyle of the population including access to modern amenities. The lifestyle changes have affected levels of physical activity and have also included introduction of a range of processed foods. New foods and food ingredients have been introduced to the diet in this region to varying extents. Similar to populations in many developing countries, the populations of EMR countries are experiencing a nutrition transition characterized by replacement of traditional diets with diets higher in fat, refined sugar, and processed foods. These changes in dietary patterns and lifestyles have been associated with high prevalence of diet-related chronic diseases such as obesity, cardiovascular diseases, type 2 diabetes mellitus, cancer, and osteoporosis in the EMR countries. 

The paper by A. C. Fahed et al. presents the relationship between diet, genetics, and diseases in the Middle East and North Africa (MENA). There are different mechanisms in this region through which diet-genetics interaction affects non communicable diseases metabolism and micronutrient pathways and contributes to diseases, including deficiencies in.calcium, iron, folate, and vitamins D, C, and E. The paper by E. S. Al-Eisa and H. I. Al-Sobayel is on physical activity and health beliefs among Saudi women. The findings revealed a high level of inactivity among Saudi women in reference to the international recommendation for minimum activity, and there was significant association between physical activity and health beliefs. The paper by E. Bakhshi et al. addresses the association between age and weight gain in Iranian women. Age was directly associated with overweight and obesity among these group. Women aged 20–40 years have highest weight compared to other age groups.

The paper by D. El Khoury and S. Antoine-Jonville assesses the prevalence of nutritional supplements intake and potential influencing factors among people excercising in gyms in Beirut, Lebanon. The intake of nutritional supplements was found to be 36.3%, with weak presence of medical supervision. Age and sex were associated with patterns of nutritional supplements. The paper by A. A. Al-Nuaim et al. studies the prevalence of physical activity and sedentary behaviours relative to obesity in Saudi adolescents. It was found that adolescents living in rural desert were less physically active than those living in urban or rural farm environment. The results also revealed that male adolescents were more active than females and physical activity levels declined with age.

The paper by A. O. Musaiger et al. focuses on establishing food based dietary guidelines (FBDG) for Arab countries in the Gulf. This is the first attempt to establishing such guidelines in the region. The paper summarizes the steps taken to develop these guidelines. The FBDG consist of 14 simple and practical advices to prevent and control nutrition-related diseases in these countries. The paper by S. Mehdad et al. assesses obesity indicators and fasting blood glucose among adolescents in Morocco. Body mass index (BMI) and waist circumference (WC) were closely associated with fat mass and body fat. However, these associations depended on geneder and weight status, and Body mass index (BMI) may provide a bett proxy estimate of overall obesity than waist circumference (WC). However, both of them are useful tools to identify adolescents at increased risk of developing excess body fat and high level of fasting blood glucose.

The papers in this special issue provide useful information on change in lifestyle and nutrition status in the Eastern Mediterranean countries. Information provided support actions needed to promote healthy lifestyle and healthy eating among children, adolescents, and adults to prevent and control of nutrition-related diseases. We hope that this special issue will stimulate other investigators to carry out further studies on changes in lifestyle and patterns of diseases in this Region.

*Abdulrahman O. Musaiger* *Abdulrahman O. Musaiger* *Hazzaa M. Al-Hazzaa* *Hazzaa M. Al-Hazzaa* *Hamed R. Takruri* *Hamed R. Takruri* *Najat Mokhatar* *Najat Mokhatar*

